# A new method and device of aligning patient setup lasers in radiation therapy

**DOI:** 10.1120/jacmp.v17i1.5527

**Published:** 2016-01-08

**Authors:** Ui‐Jung Hwang, Kwanghyun Jo, Young Kyung Lim, Jung Won Kwak, Sang Hyoun Choi, Chiyoung Jeong, Mi Young Kim, Jong Hwi Jeong, Dongho Shin, Se Byeong Lee, Jeong‐Hoon Park, Sung Yong Park, Siyong Kim

**Affiliations:** ^1^ Department of Radiation Oncology National Medical Center Seoul Korea; ^2^ Proton Therapy Center, National Cancer Center Goyang Gyeonggi Korea; ^3^ Department of Radiation Oncology Asan Medical Center Seoul Korea; ^4^ Department of Radiation Oncology Korea Cancer Center Hospital Seoul Korea; ^5^ Proton Therapy Center, McLaren Cancer Institute Flint MI USA; ^6^ Department of Radiation Oncology Virginia Commonwealth University Richmond VA USA

**Keywords:** patient setup, laser alignment, radiation isocenter, optical alignment, star shot

## Abstract

The aim of this study is to develop a new method to align the patient setup lasers in a radiation therapy treatment room and examine its validity and efficiency. The new laser alignment method is realized by a device composed of both a metallic base plate and a few acrylic transparent plates. Except one, every plate has either a crosshair line (CHL) or a single vertical line that is used for alignment. Two holders for radiochromic film insertion are prepared in the device to find a radiation isocenter. The right laser positions can be found optically by matching the shadows of all the CHLs in the gantry head and the device. The reproducibility, accuracy, and efficiency of laser alignment and the dependency on the position error of the light source were evaluated by comparing the means and the standard deviations of the measured laser positions. After the optical alignment of the lasers, the radiation isocenter was found by the gantry and collimator star shots, and then the lasers were translated parallel to the isocenter. In the laser position reproducibility test, the mean and standard deviation on the wall of treatment room were 32.3±0.93 mm for the new method whereas they were 33.4±1.49 mm for the conventional method. The mean alignment accuracy was 1.4 mm for the new method, and 2.1 mm for the conventional method on the walls. In the test of the dependency on the light source position error, the mean laser position was shifted just by a similar amount of the shift of the light source in the new method, but it was greatly magnified in the conventional method. In this study, a new laser alignment method was devised and evaluated successfully. The new method provided more accurate, more reproducible, and faster alignment of the lasers than the conventional method.

PACS numbers: 87.56.Fc, 87.53.Bn, 87.53.Kn, 87.53.Ly, 87.55.Gh

## I. INTRODUCTION

An accurate and reproducible patient positioning is required in modern radiation therapy because the precise delivery of radiation to a patient depends on the accuracy of patient setup. Even though advanced imaging devices, such as the electronic portal imaging device (EPID), kilovoltage (kV) radiography or fluoroscopy, kV computed tomography (CT), cone beam CT, megavoltage CT, and even magnetic resonance imaging (MRI) are used for accurate patient setup, ultimately, the first step in patient alignment generally relies on the lasers in the treatment room. Therefore, the lasers must be aligned precisely to the radiation isocenter. According to the report provided by the American Association of Physicists in Medicine (AAPM) Task Group 142, the localizing lasers should be aligned to within ±2 mm of radiation isocenter for non intensity modulated radiation therapy (IMRT), ±1 mm for IMRT, and less than ±1 mm for stereotactic radiosurgery (SRS) on a monthly basis.^(1)^

Identifying the radiation isocenter, obviously, is important because the treatment room lasers need to be adjusted to intersect with the radiation isocenter. Various methods have been proposed to find the radiation isocenter.^2^, ^3^, ^4^, ^5^, ^6^, ^7^, ^8^, ^9^ One of the most common and traditional ways for finding the radiation isocenter is to expose a radiation‐sensitive film with a star shot pattern.^2^, ^3^ Some authors have used an EPID together with an automated analysis software ^4^, ^5^ to find the radiation isocenter. For SRS, in which more accurate information on the isocenter location is required, Winston‐Lutz test has been conducted using diverse devices.^6^, ^7^, ^8^, ^9^ There is also a commercial tool to determine the radiation isocenter by taking X‐ray images of a phantom containing metallic ball bearings.^(10)^

However, defining the radiation isocenter only may not be sufficient for complete alignment of the lasers that consists of both positioning and tilting. In order to determine the laser position and tilting angle, Chang and his coworkers^(11)^ have used a plumb bob and a film, and Welsh's group^(12)^ has used a special device comprised of levels attached to the gantry head for gantry angle verification.

Although a few sophisticated methods utilizing a mirror have been proposed,^13^, ^14^, ^15^ the most commonly used method, as in the Welsh's approach, does use a level (preferably high accuracy), the light source inside the gantry head (i.e., light bulb), and the CHL in the gantry head (i.e., gantry head exit window). The level is used to determine a horizontal position of the gantry, but this approach can be very sensitive to several uncertainty sources such as possibly imperfect gantry head, wrong installation and/or movement of the light source, and human errors in reading the level. Therefore, a single trial of the sequential alignment of both lateral lasers is often not able to provide satisfactory alignment, and the same procedure needs to be repeated multiple times. This iterative alignment, obviously, is time‐consuming and inconvenient for one person to perform.

In this study, we propose a more accurate, reproducible, and straightforward alignment method of the patient‐setup lasers in radiation therapy. Moreover, it can be easily performed by one medical physicist. The proposed method consists of two steps: in the first step, all the paired lasers overlap completely by an optical method, and in the second step, the lasers are translated parallel to the radiation isocenter after finding its position with the gantry and collimator star shot patterns.

## II. MATERIALS AND METHODS

### A. Basic components of the proposed method and device for laser alignment

**Figure 1:**
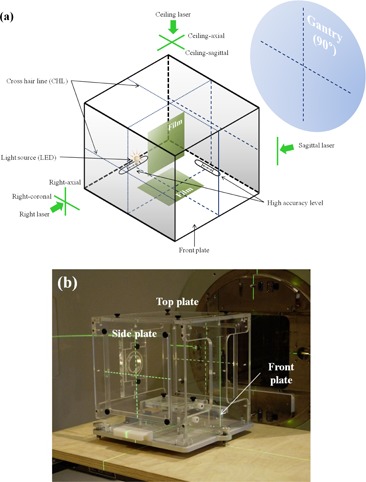
A prototype of laser aligning device: (a) schematic and (b) a photograph of the device placed on the patient table.

**Figure 2:**
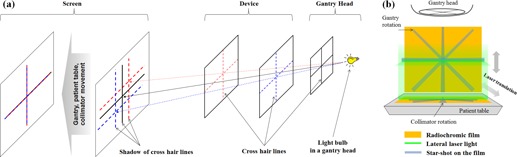
Schematic diagram of the proposed laser alignment: (a) optical alignment and (b) radiation alignment.

Our proposed method for aligning the patient‐setup lasers is realized using an in‐house–developed laser alignment device (Fig. 1), and the procedure consists of two functional steps: optical alignment and radiation alignment (Fig. 2). The device is cube‐shaped with dimensions of $30\times 30\times 30\;\;{\text{cm}}^{3}$ and contains a metal bottom plate and five transparent acrylic plates. There are a total of four CHLs inscribed, one each on every plate except the front and back (i.e., top, bottom, and two lateral sides). On the front plate, a single vertical line inscribed exists. It also contains two slots for radiochromic film insertion. One is horizontally placed near the bottom plate (horizontal slot) and the other vertically near the back plate (vertical slot). The vertical slot is movable and can be moved to the center of the device. Both film slots are rotatable about the horizontal and the vertical axis, respectively. A light‐emitting diode (LED) positioned on the longitudinal central line of the bottom plate can illuminate both the CHL of the top plate and the single vertical line of the front plate to create shadows on the ceiling and the wall facing to the front plate simultaneously. There are two high‐accuracy spirit levels installed on the bottom plate along the two orthogonal directions. The horizontality of the bottom plate is adjustable by three leveling feet. The device was manufactured with a machining accuracy of less than 20 μm and an assembly accuracy of about 0.1 mm.

As in many commonly used methods, in principle, each functional step requires three objects: a divergent beam source, a reference object, and a displayed image of the reference object. In the optical alignment, the tungsten bulb mounted inside the gantry head or the LED in the device is the divergent beam (i.e., visible‐light) source, a CHL (or the single vertical line) the object, and the shadow of the CHL the displayed image. On the other hand, in the radiation alignment, an X‐ray beam from the linear accelerator is the source, jaw or collimator the reference object, and a radiation pattern formed on the radiochromic film the displayed image.

In this study, “laser” represents either a laser generator itself or laser light emitted from the laser generator. “Lateral” indicates either right or left of which direction is defined based on a conventional room‐coordinate system when a patient is in head‐first (toward the gantry) and supine position. Each lateral (i.e., right or left) laser generator provides two laser lights: the axial (i.e., vertical) and the coronal (i.e., horizontal). The ceiling laser, obviously located on the ceiling, creates ceiling‐axial and ceiling‐sagittal laser lights. All the names of the planes and lasers are described in Fig. 1.

### B. Laser alignment workflow

**Figure 3:**
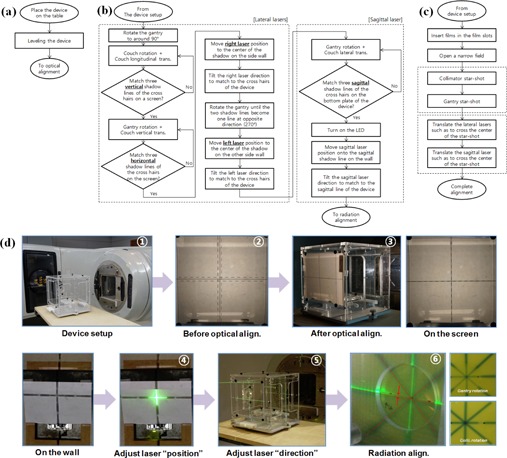
The laser alignment workflow: (a) device setup, (b) optical alignment, (c) radiation alignment procedures, and (d) a summarized photo illustration of the workflow of a lateral laser alignment.

First, the fabricated device is placed horizontally on the patient table close to the isocenter position. The horizontality of the device is critical and can be achieved using two orthogonal high‐accuracy spirit levels and three leveling feet on the bottom plate. In the next step, optical alignment for the lasers is carried out as described in the next subsection (B.1), and then radiation alignment is performed at the last (see subsection B.2). Figure 3 shows the flow charts of the overall alignment procedure ((a), (b), and (c)) and photo illustrations ((d)).

#### B.1 Optical alignment

The goal of the optical alignment of two lateral lasers is to make them coincide while keeping their horizontality. After the device is placed around the isocenter, main room light is turned off and light field from the gantry head is projected with the collimators fully open. The gantry is rotated to around a horizontal position (i.e., 90° or 270°) to obtain three shadows of three CHLs (one on the gantry head and two on the lateral plates of the device) on a screen (a flat paper used in this study), as shown in Fig. 2(a). In order to make all three vertical shadow lines coincide on the screen, longitudinal translation and rotation of the patient table are necessary and this is obtained as follows:

1. Adjust the collimator to be parallel to the CHLs of the device.
2. Adjust the table angle and longitudinal position so that the vertical line of the gantry CHL is positioned in the middle of the two vertical lines of the device.
3. Rotate the table until all of three vertical shadow lines coincide.

In the same manner, all of three horizontal shadow lines can be matched into one by both vertical translation of the table and gantry rotation. That is, the gantry angle and the table's vertical position are adjusted so that the horizontal line of the gantry CHL is positioned in the middle of the two horizontal lines of the device, and then the gantry is rotated until the three horizontal shadow lines become one. After the matching is done on the screen, the screen is removed so that the CHL shadow can be projected on the wall of the treatment room. And the laser mounted on the wall is linearly moved so that the center of the laser light coincides with the center of the CHL shadow. Then, the laser light is tilted horizontally and/or vertically towards the CHLs on the device.

For the alignment of the laser on the other side, the gantry is rotated backward about 180°, the screen is placed in the opposite side, and fine adjustment of gantry angle is performed until the only two CHLs in the device coincide with each other (i.e., not including the CHL in the gantry head). This is to avoid possible error caused by a potential positioning imperfection of the light bulb inside the gantry head. After the matching, the screen is removed, and the other lateral laser is adjusted in the same way described before.

Now, the ceiling‐axial laser can be aligned using two CHLs on the top and bottom plates of the device. The ceiling‐axial laser is aligned to pass through the two lines forming an axial plane.

Before aligning the sagittal laser and the ceiling‐sagittal laser, the lateral position of the device should be adjusted first. With a gantry angle of near zero degrees, the shadows of the CHLs in the gantry head and top plate are projected onto the bottom plate where another CHL is inscribed. Similarly to the lateral laser alignment, gantry rotation and lateral translation of the patient table are necessary to make the all longitudinal shadow lines overlap together. At this point, the gantry is at zero degrees accurately, and both the center of the device and LED are on a sagittal plane passing through the optical isocenter. Now, the gantry is rotated to clear the view of the ceiling laser and the LED is used as the light source to create a CHL shadow on both the ceiling and wall of sagittal laser, and the sagittal laser and the ceiling‐sagittal laser are placed on the corresponding shadow line. After adjusting the positions of the lasers, their directions are also tilted towards the CHLs on the device.

#### B.2 Radiation alignment

After all of the lasers are aligned optically, an alignment with the radiation isocenter is performed. Note that the optical isocenter might not agree with the radiation isocenter. In the radiation alignment process, the lasers are translated parallel to the radiation isocenter. The radiation isocenter can be found by two star shots, one with the collimator and the other with the gantry, made on two radiochromic films inserted into two orthogonal film slots in the device (Fig. 2(b)). A narrow radiation field of 0.5 cm×10 cm is used for the collimator star shot at several collimator angles, and the same field is used for the gantry star shot at several gantry angles. Jaw (i.e., collimator) calibration is a prerequisite for this process.

All axial lasers should pass through the center of the collimator star shot, and all coronal lasers should pass through the center of the gantry star shot while all sagittal lasers should pass through both centers. Therefore the parallel translation of each laser is the final step in the whole alignment process.

### C. Evaluation of the method

The reproducibility, accuracy, and efficiency of the proposed laser alignment were evaluated and compared with the conventional method, which used the light source and CHL in the gantry head at two horizontal gantry angles (90° and 270°) determined by a level. We also investigated the influence of the light source positional error. The alignment reproducibility was quantified by the standard deviation of the shadow height measured on the wall repeatedly, the alignment accuracy was quantified by the difference of the shadow heights on both side walls, and the alignment efficiency was also quantified by the time taken to perform the alignment. The shadow heights were read from a reference height which could be defined by a long transparent hosepipe filled with water. After removing air bubbles from water and fixing both ends of the hosepipe to the both side walls, the water levels were marked on graph papers attached to the walls. The water level was determined with an accuracy of less than 0.5 mm in height through repeated measurements. This experiment was carried out in a treatment room where a linac (Varian Medical Systems, Palo Alto, CA) and a set of lasers (LAP, Luneburg, Germany) were installed.

#### C.1 Alignment reproducibility

**Figure 4:**
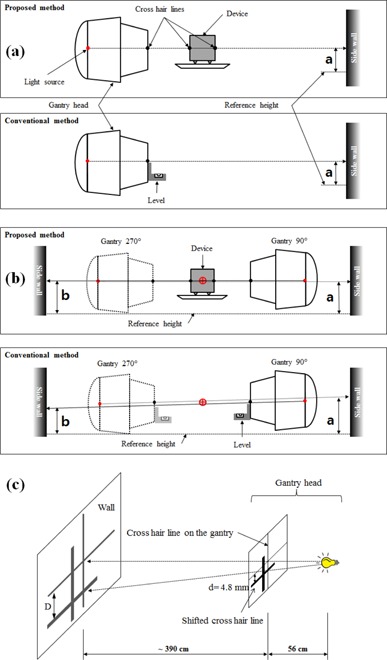
Measurement of (a) reproducibility, (b) accuracy, and (c) effect of light source position error in the laser alignment.

In order to measure the alignment reproducibility, after aligning the gantry horizontally according to each method (the conventional and proposed method), the shadow height (“a” value shown in Fig. 4(a)) was measured 30 times by three individuals. After reading the shadow height, the aligned gantry and patient table were reset to an arbitrary position to start another alignment. In the alignment using the conventional method, two tests, one with a high‐accuracy level (0.44 mm/1,000 mm) and the other with a low‐accuracy level (N/A) were used for comparison, respectively.

#### C.2 Alignment accuracy

A shadow height on a side wall was defined as “a” from the reference height, and the other shadow height on the opposite side wall was defined as “b”. Since the lasers on both side walls should be positioned at the same level, there is no height difference in the well‐aligned lateral lasers. Therefore, their difference, b – a, could be used as a measure of the alignment accuracy and it was compared between the proposed method and the conventional method (Fig. 4(b)).

#### C.3 Alignment efficiency

The alignment efficiency is about how much time is taken to perform the alignment and whether one person can do the job effectively. Since the latter is not easy to quantify we will discuss it qualitatively. The time taken to align the lateral lasers was measured for the proposed method, as well as the conventional method. For the proposed method, only optical alignment time was included for a fair comparison. It was measured in six treatment rooms in four hospitals.

#### C.4 Effect of light source position error

Light field from the gantry head is used to align the lasers in both the proposed and conventional methods. If there is a position error of the light source, then it can lead to a wrong positioning of the laser. In this section, the effect of light source position error on the laser alignment was also compared between both methods. However, it is not practical to produce position errors artificially. Instead, thus, a fake CHL sheet was attached on the gantry head with a lateral shift from the beam axis to simulate a CHL shadow that would have been generated with a position error of the light source (Fig. 4(c)). The distance from the light source to the CHL plate was 56 cm, and the distance from the CHL to the wall was about 390 cm (Fig. 4(c)). According to the triangle rule, the magnification of the shift distance was about eight times in the wall.

After completing the optical alignment using the conventional method, the height difference between the shadows created by the original and fake CHLs was measured on a side wall. The same measurement was performed for the proposed alignment method, and their results were compared. For this test, the shift distance was 4.8 mm from the beam axis along the in‐plane. In another test where the shift distance was 3.0 mm, the optical alignment was made in both walls and the difference between walls was evaluated.

## III. RESULTS

### A. Alignment reproducibility

**Figure 5:**
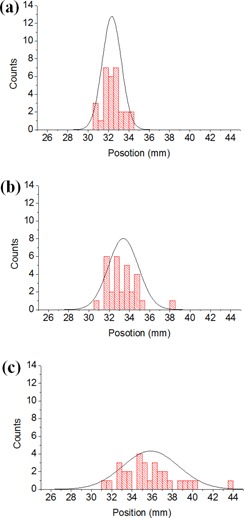
Alignment reproducibility of the laser positions for (a) the proposed method and conventional methods using (b) high‐ and (c) low‐accuracy levels: (a) m=32.3,σ=0.93, (b) m=33.4,σ=1.49, and (c) m=35.9,σ=2.76, where m and σ denote the mean value and standard deviation of the laser position in mm units, respectively. The resolutions of the levels used in proposed and conventional (with a high‐accuracy level) methods were 0.05 mm/1,000 mm and 0.44 mm/1,000 mm, respectively.

The position of the CHL's shadow was repeatedly measured on a side wall, and their distribution is presented in Fig. 5. For the proposed method, the mean and standard deviation (SD) of the positions on the wall were 32.3±0.93 mm, whereas they were 33.4±1.49 mm and 35.9±2.76 mm for the conventional methods using a high‐accuracy level and a low‐accuracy level, respectively. The standard deviation was lowest for the proposed method. The difference of the mean positions was 1.1 mm between the proposed method and the conventional method using the high‐accuracy level. There was a large discrepancy, 3.6 mm, between two cases of the conventional method, with a high‐accuracy level and with a low‐accuracy level.

### B. Alignment accuracy

The standard deviation of the laser position differences, b – a, on both side walls was 1.4 mm for the proposed method, while it was 2.1 mm and 26.0 mm for the conventional methods, using a high‐accuracy level and a low‐accuracy level, respectively (see Table 1). These values are standard deviation of four measurements. The maximum value of b‐a was 1.5 mm among the measurements in the proposed method. Note this value is at the wall and corresponds to approximately 0.12 mm at around ±15 cm from the isocenter.

**Table 1:** Comparison of the position differences of the aligned lasers on both side walls between the proposed and conventional methods.

		*Conventional Method (mm)*	
	*Proposed Method (mm)*	*High‐accuracy level* ^b^	*Low‐accuracy level*
b‐a^a^ (Fig. 4(b))	1.4	2.1	26.0

^a^ The value is an SD of four measurements.

^b^ A high‐accuracy level with the resolution of 0.44 mm/1,000 mm was used.

### C. Alignment efficiency

The average time for optical alignment was less than 20 minutes for our proposed method while it was about an hour for the conventional method.

### D. Effect of light source position error

With the gantry CHL shifted by 4.8 mm from its original position, in the case of the conventional method, the shift of the laser position was 36.9 mm at the wall, which was about eight times the 4.8 mm. The number eight was determined by the geometry of our treatment room. However, the proposed method showed 5.3 mm shift at the wall, which was only 0.5 mm different from the error‐simulating shift of 4.8 mm (see Table 2).

The average difference between the positions on both side walls was 0.5 mm for the proposed method when the gantry CHL was shifted by 3.0 mm from its original position. However, the difference was 48.5 mm for the conventional method (with a high‐accuracy level) (see Table 3). Note that the possible geometrical magnitude increase is 2×8=16 in this case because both walls are involved.

**Table 2:** Effect on the laser position on the wall when the gantry CHL was shifted by 4.8 mm.

		*Laser Position on the Wall* ^b^ *(mm)*		
*Alignment Method*	*CHL Position Shift (mm)*	*m*	$m^{\prime }$	$\left|m^{\prime }-m\right|$
Conventional method^a^	4.8	33.4	‐3.5	36.9 (~ 8d)
Proposed method	4.8	32.3	27.0	5.3 (~ d)

^a^ A high‐accuracy level with the resolution of 0.44 mm/1,000 mm was used.

^b^ The value was averaged over three measurements.

Note: m is for the case of no shift and m' is for the case of existing CHL position error.

**Table 3:** Effect on the laser alignment accuracy when the gantry CHL was shifted by 3.0 mm.

		*Laser Position On Both Walls* ^b^ *(mm)*		
*Alignment Method*	*CHL Position Error (mm)*	*a*	*b*	*b‐a* (Fig. 4(b))
Conventional method^a^	3	57.5	9.0	−48.5(∼2×28d)
Proposed method	3	39.3	38.8	‐0.5

^a^ A high‐accuracy level with the resolution of 0.44 mm/1,000 mm was used.

^b^ The value was averaged over three measurements.

## IV. DISCUSSION

In the laser alignment using a conventional method, the laser position is determined by a level attached to the surface of the gantry head. This approach works under two critical assumptions: 1) that the gantry head surface is flat and perpendicular to the central axis of the beam, and 2) that the light source and CHL in the gantry head are perfectly aligned. The verification of these assumptions is not an easy task and often is not included in routine laser alignments. However, there are many factors that might make the assumptions less rigorous such as imperfect assembly of the gantry head, damage of gantry head surface (either by incident or long‐term use), and displacement of the light source and/or CHL in the gantry head.

Considering such uncertainty sources, we believe that higher alignment reproducibility and accuracy can be obtained by removing the risky factors from the alignment process. For example, the new alignment device in our proposed method is just placed on the patient table and does not move through the whole alignment process, which would help performing more consistent laser alignment.

**Figure 6:**
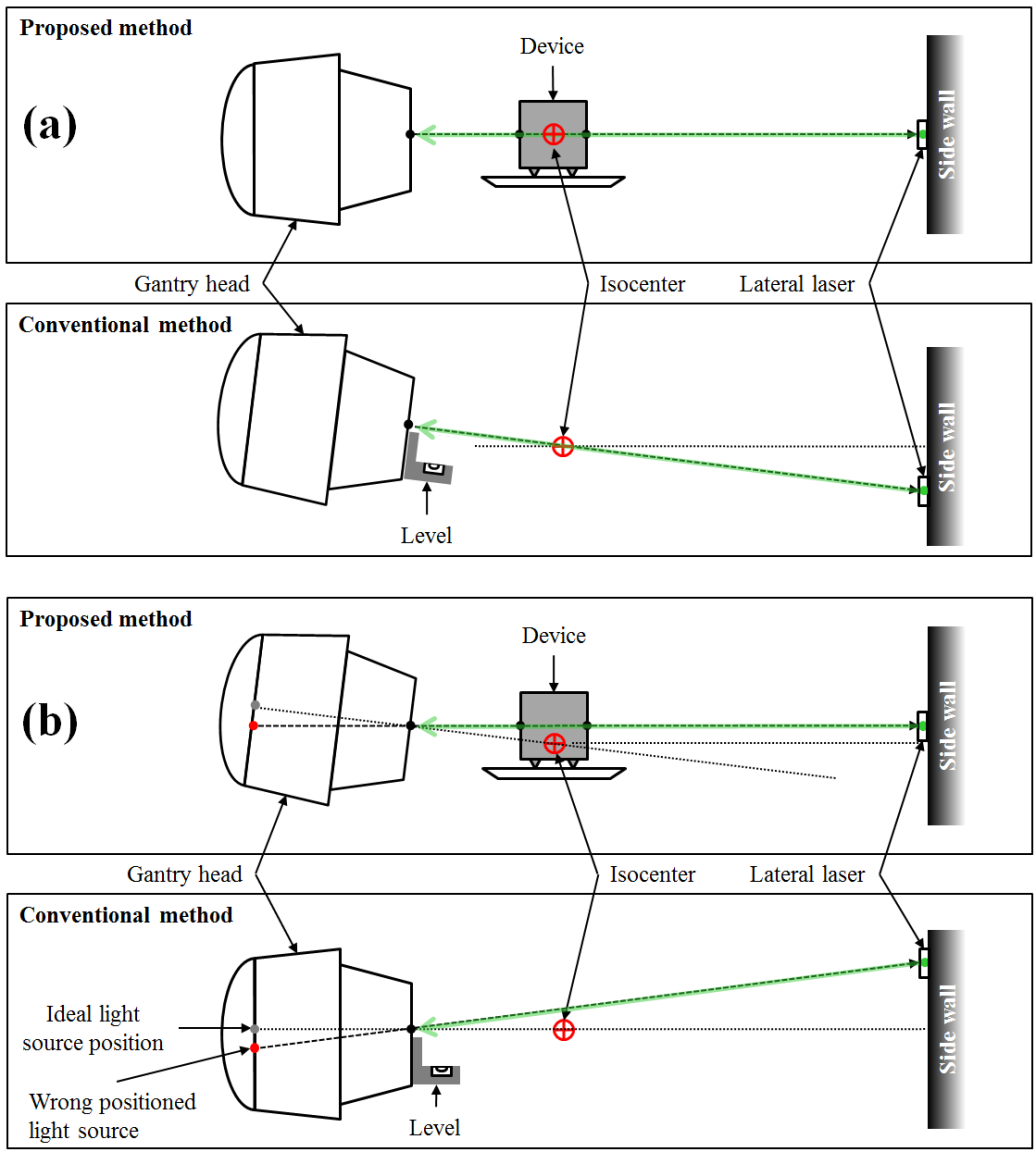
Comparison of influences of (a) imperfection of gantry head and (b) light source position error.

The accuracy of the conventional method would significantly degrade if the gantry has some imperfections, especially the light source position error as illustrated in Figure 6(a), but, to our best knowledge, no explicit test on such situations has been reported, mainly due to practical difficulty in creating similar situations. In this study, we introduced a method that mimics such condition without displacing the actual light bulb but by introducing a fake CHL in the gantry head (Fig. 4). This method was used for the evaluation of the impact of light source position error. The results of the test confirmed that the conventional method is very sensitive to light source displacement (1, 2). However, the proposed method showed behavior which was relatively less sensitive to the error of the light source position (i.e., no magnifying effect on the wall due to divergence). In addition, since the proposed method includes radiation alignment as well, any such error can be significantly mitigated.

Another advantage of the proposed method is the alignment efficiency. Since our laser alignment method is not iterative but deterministic, a straightforward alignment is achievable; thus, the required time for aligning the lasers decreases dramatically. A single medical physicist can align the lasers accurately within a half hour.

The radiation star shot takes longer, inevitably, while the optical alignment can be done quickly with high reliability. In actual practice, all the lasers including the ceiling and sagittal lasers can be checked daily using only the optical alignment method, in about five minutes.

The same lasers can also be checked more accurately using radiation fields in monthly quality assurance (QA). If they are out of the allowable tolerance range, a full alignment procedure has to be performed. If the Winston‐Lutz method is adopted in our device and an EPID is used to determine the radiation isocenter instead of the star shot method,^(4)^ more accurate alignment may be achieved since the star shot method is limited to quantify three dimensional wobble of radiation field centers.^(5)^

Our method, not being restricted to conventional photon therapy, is applicable to proton therapy and radiation therapy simulation. We tried to align the lasers in the proton treatment room available at the National Cancer Center in Korea, where an IBA proton therapy system, Proteus 235 (IBA, Louvain‐la‐Neuve, Belgium) was installed. There were seven lasers in a gantry treatment room; three lasers on the gantry, two lasers on both lateral sides, one laser at the end of the nozzle, and one on the ceiling. The laser system differs from that of conventional photon therapy because several lasers rotate together with the gantry. But they were aligned successfully by using our device with slight modification of procedures.

With the laser alignment, our proposed device can be applied to the mechanical QA. For example, our device is applicable for gantry angle calibration because the gantry angles of 0°, 90°, and 270° can be easily found with the device. In the same way, we can also calibrate the collimator angles at 0°, 45°, 90°, 315°, and 270° with the CHLs in the device. Collimator and gantry star shots are basically conducted during the radiation alignment process. There is no need to repeat those procedures in a regular mechanical QA. Once a radiation isocenter is defined in the alignment process, our device can be a reference for checking the front pointer or optical distance indicator (ODI). Our proposed method will reduce the time for executing mechanical QA and improve work flow for medical physicists.

## V. CONCLUSIONS

We proposed and demonstrated a deterministic method to align the patient setup lasers. The method provided more accurate, more reproducible and faster alignment of the lasers than the conventional method by using radiation isocenter, absolute horizontal and vertical references, and a straightforward alignment method as well as by excluding the incomplete factors in the conventional alignment method. Laser alignment in the proposed method was not affected by any environmental conditions, such as imperfect gantry head and position error of light source or CHL in the gantry head. The developed device also has extendibility to various mechanical QAs of LINACs as well as to other treatment machines.

## ACKNOWLEDGMENTS

The authors would like to thank the reviewers for their valuable comments on this manuscript. This research was supported by a National Cancer Center Grant (NCC‐1210540) and was also supported by a National Research Foundation of Korea (NRF) grant funded by the Korea government (MSIP) (NRF‐2011‐0018931).
